# Inferring Active Noise Characteristics from the Paired Observations of Anomalous Diffusion

**DOI:** 10.3390/polym11010002

**Published:** 2018-12-20

**Authors:** Takuya Saito, Takahiro Sakaue

**Affiliations:** 1 Earthquake Research Institute, University of Tokyo, Tokyo 113-0032, Japan; 2 Department of Physics and Mathematics, Aoyama Gakuin University, Chuo-ku, Sagamihara 252-5258, Japan; sakaue@phys.aoyama.ac.jp; 3 JST, PRESTO, 4-1-8 Honcho Kawaguchi, Saitama 332-0012, Japan

**Keywords:** polymer, anomalous diffusion, nonequilibrium noise

## Abstract

Anomalous diffusion has been most often argued in terms of a position fluctuation of a tracer. We here propose the other fluctuating observable, i.e., momentum transfer defined as the time integral of applied force to hold a tracer’s position. Being a conjugated variable, the momentum transfer is thought of as generating the anomalous diffusion paired with the position’s one. By putting together the paired anomalous diffusions, we aim to extract useful information in complex systems, which can be applied to experiments like tagged monomer observations in chromatin. The polymer being in the equilibrium, the mean square displacement (or variance) of position displacement or momentum transfer exhibits the sub- or superdiffusion, respectively, in which the sum of the anomalous diffusion indices is conserved quite generally, but the nonequilibrium media that generate the active noise may manifest the derivations from the equilibrium relation. We discuss the deviations that reflect the characteristics of the active noise.

## 1. Introduction

Developments of imaging technologies on the microscopy have opened up the way to monitor molecular motions in cells and cellular nuclei. One of the most interested observables is a position x(t) of a tracer or a labeled spot, fluctuations of which are commonly quantified by asymptotics of the mean-square displacement (MSD) [1,2,3,4,5,6,7,8,9,10,11]:

(1) $$\begin{array}{c} \langle \delta x{\left(t\right)}^{2}\rangle \equiv \langle {\left\{x\left(t_{0}+t\right)-x\left(t_{0}\right)\right\}}^{2}\rangle ∼t^{\alpha^{\left(x\right)}}. \end{array}$$

Assuming a stationary process, the MSD does only depend on the lag time *t* but not on the time origin $t_{0}$, and characterized by the exponent $\alpha^{\left(x\right)}$. While a simple particle in a viscous fluid displays normal diffusion with $\alpha^{\left(x\right)}=1$, the labeled spots in the chromosomal structure create anomalous diffusion with $\alpha^{\left(x\right)}\neq 1$ and have drawn extensive attention. A rich variety of indices $\alpha^{\left(x\right)}$ has been reported [4,5,6,7,8,9,10] and thought of as a consequence of the combined factors [12,13]. A primary factor is a chain connectivity. As illustrated in the Rouse model with $\alpha^{\left(x\right)}=1/2$ [14,15,16,17], the connectivity in chain-like structure lowers index $\alpha^{\left(x\right)}$, making the subdiffusion commonplace in polymeric systems. There are several factors to affect the index $\alpha^{\left(x\right)}$—for instance, self-avoiding (SA) effects and hydrodynamic interactions (HIs) [14,18,19,20,21]. If the molecule develops some branching structure [22,23], it may also modify $\alpha^{\left(x\right)}$ [23,24,25]. In addition, as reported in experimental, numerical, or theoretical studies [1,4,5,6,7,8,9,10,11,12,13], the diffusion have been investigated in the presence of the nonequilibrium force, the so-called active noise, as a product of the metabolic activity in cells. Generally, however, it might force hard work on us to specify characteristics of the active noise in the experiments because the anomalies in the living cells are generated due to the combined factors.

One of the possible ways to identify the anomalous diffusion features generated by the active noise is to employ deviations from equilibrium relations. We here turn our attention to power–law relations in a complex background between the MSD of the tracer, and that of the fluctuating force required to keep the tracer in a fixed position. While the former is familiar in a standard stochastic analysis, the latter quantity is not well documented in literature, and thus requires some explanation. Let us consider the situation, in which the position of a tracer is fixed in space. To realize it, the force f(t) is necessary, which is a fluctuating quantity. From the observed force f(t), we consider the diffusion of momentum transfer $p\left(t\right)=\int_{0}^{t}ds\;f\left(s\right)$ defined as the integral of f(t) over time:

(2) $$\begin{array}{c} \langle \delta p{\left(t\right)}^{2}\rangle ∼t^{\alpha^{\left(p\right)}}. \end{array}$$

For a simple Brownian particle, the ratio of the momentum transfer to the position displacement is a constant (friction coefficient). One thus immediately finds $\alpha^{\left(p\right)}=1$. This normal diffusion behavior may, however, break down for more complicated situations, where viscoelastic memory effect plays a role. As a simple example, consider a tagged monomer in a Rouse polymer. The analysis based on the generalized Langevin equation (GLE) leads to the superdiffusion with $\alpha^{\left(p\right)}=3/2$ [26,27]. Since the position displacement and the momentum transfer are conjugate to each other with respect to Hamiltonian, one expects that there may be some relation between $\alpha^{\left(x\right)}$ and $\alpha^{\left(p\right)}$. Indeed, from the analysis of the GLE with the fluctuation–dissipation relation, one can verify the following sum relation generally holds in thermal equilibrium [26]:

(3) $$\begin{array}{c} \alpha^{\left(x\right)}+\alpha^{\left(p\right)}=2. \end{array}$$

It is stressed that Equation (3) holds as long as it is in equilibrium. On the other hand, the nonequilibrium environments driven by the the active noises do not necessarily ensure the asymptotic relation Equation (3). One might then think what can be extracted from combining the observations of x(t) and p(t), and also if the deviations from the equilibrium provide further useful information, e.g., to understand the chromatin. Indeed, the nonequilibrium force is often monitored to investigate polymer structures [28] or mechanical characteristics in molecular motors [29,30,31] such as actomyosin, kinesin, and polymerase, etc., the processes of which the fluctuations are intrinsically involved in.

This article proposes the approach to infer the noise characteristics by monitoring the paired observables x(t), p(t) for the tagged monomer in the polymer. Section 2 begins with introducing the key concept for the paired observables by taking a simple probe that does not have a degree of internal freedom. Next, Section 3 reviews how the internal configurations create the anomalous diffusion of the position x(t) for the Rouse polymer in the presence of thermal or active noise through the mode analysis. Besides the chain connectivity, Section 3.1.2 incorporates the nonlinearities like the SA effects, HIs or the branchings into the polymer model. Then, Section 3.2 discusses the other observable p(t) and summarizes the deviations from Equation (3). In addition, Section 4.1 briefly lists the remarkable results for the viscoelastic media (see Appendix A for more details). Section 4.2 considers more generalized noise and develops the arguments about how the deviations from Equation (3) may be utilized to narrow down the candidates for the noise. Then, Section 4 presents a discussion, and Section 5 concludes this study.

## 2. Paired Observables in Simple Probe

To illustrate the idea of complementary diffusion analysis, we first consider a system, in which a simple probe is embedded in a viscoelastic medium, and subjected to thermal and active noises.

### 2.1. Position Fluctuation in Force Free Protocol

The time evolution of the probe position x(t) (of, say, *x* component), obeys the following overdamped generalized Langevin equation

(4) $$\begin{array}{c} \int_{0}^{\infty }G\left(t-s\right)\dot{x}\left(s\right)ds=\xi_{th}\left(t\right)+\xi_{A}\left(t\right), \end{array}$$

which describes a balance between the viscoelastic force (left-hand side) and the random forces (right-hand side) acting on the probe with the dot $\dot{\left(\;\right)}$ denoting the time derivative. Here, while the auto-correlation of the thermal noise $\xi_{th}\left(t\right)$ is related with the memory kernel G(t) as

(5) $$\begin{array}{c} k_{B}TG\left(t-t^{\prime }\right)=\langle \xi_{th}\left(t\right)\xi_{th}\left(t^{\prime }\right)\rangle , \end{array}$$

no such a restriction exists for the active noise $\xi_{A}\left(t\right)$. We assume that the memory kernel G(t) takes a power–law form

(6) $$\begin{array}{c} G\left(t\right)=\Gamma_{\lambda }\;t^{-\lambda } \end{array}$$

with the stress relaxation exponent λ≤1 and the generalized friction constant $\Gamma_{\lambda }$, and the temporal correlation of the active noise decays exponentially

(7) $$\begin{array}{c} \langle \xi_{A}\left(t\right)\xi_{A}\left(s\right)\rangle =A\;e^{-|t-s|/\tau_{A}}. \end{array}$$

With an additional assumption that there is no correlation between thermal and active noises, MSD of the probe is represented as the sum of these two contributions $\langle \delta x{\left(t\right)}^{2}\rangle ={\langle \delta x{\left(t\right)}^{2}\rangle }_{th}+{\langle \delta x{\left(t\right)}^{2}\rangle }_{A}$, which are calculated as

(8) $$\begin{array}{c} {\langle \delta x{\left(t\right)}^{2}\rangle }_{th}≃\frac{k_{B}T}{\Gamma_{\lambda }}t^{\lambda }, \end{array}$$

(9) $$\begin{array}{c} {\langle \delta x{\left(t\right)}^{2}\rangle }_{A}≃\left\{\begin{array}{cc} \frac{A}{\Gamma_{\lambda }^{2}}t^{2\lambda }, & (t\ll \tau_{A}),\\ \frac{A\tau_{A}}{\Gamma_{\lambda }^{2}}t^{2\lambda -1}, & (t\gg \tau_{A}). \end{array}\right. \end{array}$$

**Figure 1:**
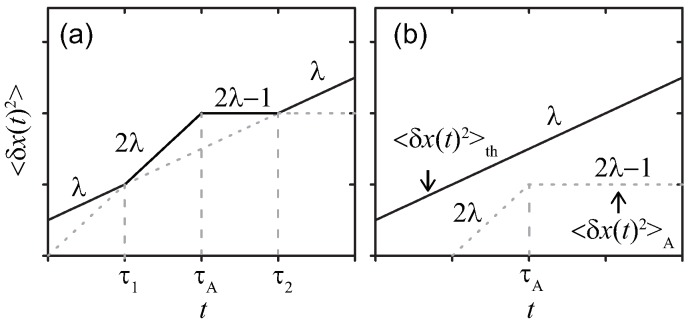
Sketches of MSDs $\langle \delta x{\left(t\right)}^{2}\rangle$ for a simple probe in a viscoelastic media at double-logarithmic scales. The active noise with (**a**) strong $k_{B}TG\left(\tau_{A}\right)<A$, or (**b**) weak $k_{B}TG\left(\tau_{A}\right)>A$ magnitude, respectively. The parameters are chosen as λ=0.5, $\tau_{A}/\tau_{1}=10.0$, and $\tau_{2}/\tau_{1}={\left(\tau_{A}/\tau_{1}\right)}^{1/(1-\lambda )}=100.0$.

The temporal profiles are drawn in Figure 1, where two characteristic time scales $\tau_{1}≃{\left(\Gamma_{\lambda }k_{B}T/A\right)}^{1/\lambda }$ and $\tau_{2}≃{\left[A\tau_{A}/\left(\Gamma_{\lambda }k_{B}T\right)\right]}^{1/(1-\lambda )}=\tau_{1}{\left(\tau_{A}/\tau_{1}\right)}^{1/(1-\lambda )}$ are determined by comparing ${\langle \delta x{\left(t\right)}^{2}\rangle }_{th}$ and ${\langle \delta x{\left(t\right)}^{2}\rangle }_{A}$. As shown in Figure 1a, the effect of the active noise manifests when the condition $k_{B}TG\left(\tau_{A}\right)<A$ is satisfied in the time range $\tau_{1}<t<\tau_{2}$. Note that the exponent 2λ−1 becomes negative for λ<0.5; hence, Equation (9) implies that, in this case, the MSD exhibits a plateau in the time interval $\tau_{A}<t<\tau_{2}^{*}$, where $\tau_{2}^{*}≃\tau_{1}{\left(\tau_{A}/\tau_{1}\right)}^{2}$ determined from the condition $A\tau_{A}^{2\lambda }/\Gamma_{\lambda }^{2}={\langle \delta x{\left(\tau_{2}^{*}\right)}^{2}\rangle }_{th}$.

### 2.2. Momentum Fluctuation in Position Fixed Protocol

Now, let us consider the situation, where the probe position x(t) is fixed by applying the external force f(t). Since $\dot{x}\left(t\right)=0$, Equation (4) is modified as

(10) $$\begin{array}{c} \dot{p}\left(t\right)=\xi_{th}\left(t\right)+\xi_{A}\left(t\right), \end{array}$$

where $p\left(t\right)=\int_{0}^{t}f\left(s\right)ds$ is the momentum transferred to the probe during the time interval t∈[0,t] due to the external force to fix the probe position, and the thermal and active noises are the same as before with their temporal correlations given by Equations (5) and (7). By plotting p(t) as a function of time, one obtains an erratic time evolution with its ensemble average zero. Just as in the case of position fluctuation, the MSD of the momentum defined as $\langle \delta p{\left(t\right)}^{2}\rangle$ (Although “D” of MSD conventionally stands for the displacement, $\langle \Delta p{\left(t\right)}^{2}\rangle$ for the momentum transfer is referred to as the MSD) is represented as a sum of thermal and active contributions, i.e., $\langle \delta p{\left(t\right)}^{2}\rangle ={\langle \delta p{\left(t\right)}^{2}\rangle }_{th}+{\langle \delta p{\left(t\right)}^{2}\rangle }_{A}$ with

(11) $$\begin{array}{c} {\langle \delta p{\left(t\right)}^{2}\rangle }_{th}≃\Gamma_{\lambda }k_{B}Tt^{2-\lambda }, \end{array}$$

(12) $$\begin{array}{c} {\langle \delta p{\left(t\right)}^{2}\rangle }_{A}≃\left\{\begin{array}{cc} At^{2}, & (t\ll \tau_{A}),\\ A\tau_{A}t, & (t\gg \tau_{A}). \end{array}\right. \end{array}$$

**Figure 2:**
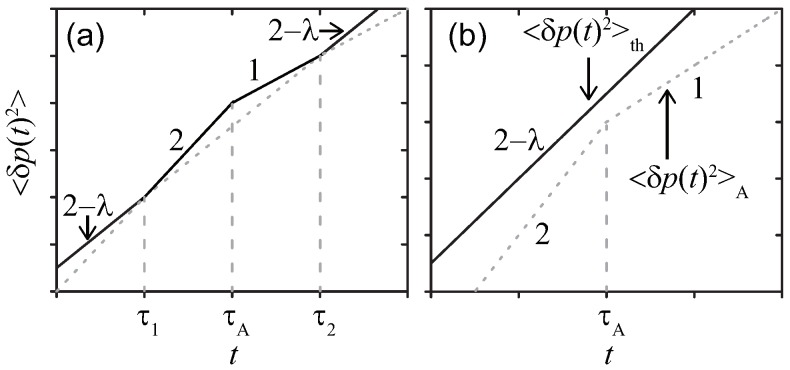
Sketches of MSDs $\langle \delta p{\left(t\right)}^{2}\rangle$ for a simple probe in a viscoelastic media at double-logarithmic scales. The active noise with (**a**) strong $k_{B}TG\left(\tau_{A}\right)<A$, or (**b**) weak $k_{B}TG\left(\tau_{A}\right)>A$ magnitude, respectively. The same parameters as those in Figure 1 are adopted.

The temporal evolutions are drawn in Figure 2. Again, the effect of the active noise manifests when the condition $k_{B}TG\left(\lambda \right)<A$ is satisfied in the time range $\tau_{1}<t<\tau_{2}$.

### 2.3. Sum of MSD Exponents

Suppose one observed the tracer position in a viscoelastic active medium and obtained the MSD in a certain interval of time scale to find the MSD exponent $\alpha^{\left(x\right)}=0.8$. Without other information, it would be difficult to infer whether this is an active diffusion in the time range $\tau_{1}<t<\tau_{A}$ with λ=0.4 or in the time interval $\tau_{A}<t<\tau_{2}$ with λ=0.9, or a thermal diffusion in the medium with λ=0.8. This difficulty is solved when one can measure the momentum fluctuation in the fixed position protocol. In particular, we find the relation $\alpha^{\left(x\right)}+\alpha^{\left(p\right)}=2$ holds whenever the thermal noise dominates the process, where the MSD exponents are defined as $\langle \delta x{\left(t\right)}^{2}\rangle ∼t^{\alpha^{\left(x\right)}}$ in force free protocol and $\langle \delta p{\left(t\right)}^{2}\rangle ∼t^{\alpha^{\left(p\right)}}$ in position fixed protocol. This relation is, however, violated when the effect of the active noise takes over, which is the case in the time interval $\tau_{1}<t<\tau_{2}$ under the condition $k_{B}TG\left(\lambda \right)<A$. Specifically, we find

(13) $$\begin{array}{c} \alpha^{\left(x\right)}+\alpha^{\left(p\right)}=\left\{\begin{array}{cc} 2\lambda +2, & (\tau_{1}<t<\tau_{A}),\\ 2\lambda , & (\tau_{A}<t<\tau_{2}). \end{array}\right. \end{array}$$

If λ<0.5, the latter relation is replaced by

(14) $$\begin{array}{c} \alpha^{\left(x\right)}+\alpha^{\left(p\right)}=\left\{\begin{array}{cc} 1, & (\tau_{A}<t<\tau_{2}^{*}),\\ \lambda +1, & (\tau_{2}^{*}<t<\tau_{2}). \end{array}\right. \end{array}$$

## 3. Paired Observables in Tagged Monomer

From Section 3, we turn to the paired anomalous diffusion of a tagged monomer in a polymer on the basis of the mode analyses. The intrinsic difference from the simple probe is the internal degree of freedom, which may produce the anomalous diffusion by itself. As a benchmark, Section 3.1.1 first illustrates the Rouse model in a viscous fluid to show how anomalous diffusion of tagged monomer’s position arises from the interplay between chain connectivity and thermal or active noise.

### 3.1. Position Fluctuations

#### 3.1.1. Rouse Polymer

Consider here a linear polymer composed of *N* monomers. The index *n* represents the material coordinate labeled from the edge, with which, for example, $x_{n}\left(t\right)$ denotes *n*th monomer’s position. Note that, if the subscript *n* drops like x(t), it corresponds to the tagged monomer’s variable. Equation of motion for the Rouse model in the viscous media is written as:

(15) $$\begin{array}{c} \gamma \frac{\partial x_{n}\left(t\right)}{\partial t}=k\frac{\partial^{2}x_{n}\left(t\right)}{\partial n^{2}}+f_{n}\left(t\right)+\xi_{n}^{\left(x\right)}\left(t\right), \end{array}$$

which represents the force balance at the overdamped time scale. The left side denotes the drag force due to viscous media. On the right side, $k\partial^{2}x_{n}\left(t\right)/\partial n^{2}$, $f_{n}\left(t\right)$ or $\xi_{n}^{\left(x\right)}\left(t\right)$ denotes the elastic force with the spring constant *k*, the external force, or the random force with the zero mean, respectively.

Letting the polymer free or exerting the constant external force on the tagged monomer, we monitor the tagged monomer’s position x(t) as the fluctuating observable. The MSD of $x_{n}\left(t\right)$ is determined according to the statistics of $\left\{\xi_{n}^{\left(x\right)}\left(t\right)\right\}$, which is composed of thermal and active components. As before (see Section 2), the active component is assumed to have the exponential temporal correlation, while the thermal component obeys the fluctuation–dissipation relation:

(16) $$\begin{array}{c} \langle \xi_{n}^{\left(x\right)}\left(t\right)\xi_{n^{\prime }}^{\left(x\right)}\left(t^{\prime }\right)\rangle =\left[2\gamma k_{B}T\delta \left(t-t^{\prime }\right)+Ae^{-|t-t^{\prime }|/\tau_{A}}\right]\delta_{nn^{\prime }}. \end{array}$$

To proceed further, converting into the mode space is useful. The normal mode $X_{q}\left(t\right)$ and the transform are defined as

(17) $$\begin{array}{c} X_{q}\left(t\right)=\int_{0}^{N}dn\;x_{n}\left(t\right)h_{q,n}\Leftrightarrow x_{n}\left(t\right)=\sum_{q\geq 1}X_{q}\left(t\right)h_{q,n}^{†}, \end{array}$$

with

(18) $$\begin{array}{c} h_{q,n}=\frac{1}{N}\cos\left(\frac{\pi qn}{N}\right),\;h_{q,n}^{†}=2\cos\left(\frac{\pi qn}{N}\right). \end{array}$$

Our interest is here the internal configuration fluctuations. This article looks only at the internal modes for q≥1 by focusing on the temporal regime within the longest relaxation time of the polymer, while not dealing with the center of mass mode q=0. Using Equation (17), Equation (15) of motion is rephrased by

(19) $$\begin{array}{c} \gamma_{q}\frac{dX_{q}\left(t\right)}{dt}=-k_{q}X_{q}+F_{q}\left(t\right)+\Xi_{q}^{\left(x\right)}\left(t\right), \end{array}$$

with the coefficients

(20) $$\begin{array}{c} \gamma_{q}=\gamma ,\;k_{q}=k{\left(\frac{q}{N}\right)}^{2}, \end{array}$$

and also Equation (17) is as

(21) $$\begin{array}{c} \langle \Xi_{q}^{\left(x\right)}\left(t\right)\Xi_{q^{\prime }}^{\left(x\right)}\left(t^{\prime }\right)\rangle =\frac{\gamma_{q}}{N\gamma }\delta_{qq^{\prime }}\Phi \left(t-t^{\prime }\right), \end{array}$$

with the time-dependent factors

(22) $$\begin{array}{c} \Phi \left(t-t^{\prime }\right)=2\gamma k_{B}T\delta \left(t-t^{\prime }\right)+Ae^{-|t-t^{\prime }|/\tau_{A}}. \end{array}$$

We can find the analytical solution to Equation (19) since it is a linear stochastic equation. Superimposing all the modes $\left\{X_{q}\left(t\right)\right\}$ with Equation (17), we extract the major contribution to determine the MSD scalings as

(23) $$\begin{array}{ccc} \langle \delta x{\left(t\right)}^{2}\rangle & ≃ & \int_{0}^{t}ds\int_{0}^{t}ds^{\prime }\;\sum_{q,q^{\prime }}\frac{\langle \Xi_{q}^{\left(x\right)}\left(s\right)\Xi_{q^{\prime }}^{\left(x\right)}\left(s^{\prime }\right)\rangle }{\gamma_{q}\gamma_{q^{\prime }}}\\ & & \times e^{-\left(k_{q}/\gamma_{q}\right)\left(t-s\right)}e^{-\left(k_{q^{\prime }}/\gamma_{q^{\prime }}\right)\left(t-s^{\prime }\right)}h_{q,n}^{†}h_{q^{\prime },n}^{†}\\ & ≃ & \int_{0}^{t}ds\int_{0}^{t}ds^{\prime }\;\frac{1}{N\gamma^{2}}{\left(\frac{\gamma }{k}\right)}^{1/2}{\left|2t-s-s^{\prime }\right|}^{-1/2}\Phi \left(s-s^{\prime }\right), \end{array}$$

where we take the continuum limit of *q* to employ the integral formula:

(24) $$\begin{array}{c} \int_{0}^{\infty }dx\;x^{b-1}e^{-ax^{\theta }}=\Gamma \left(b/\theta \right)a^{-b/\theta }/\theta , \end{array}$$

where Γ(·) denotes the gamma function, and the parameters are *a*, *b* and c>0. In the present case b=1, θ=2, the formula reduces to an ordinary Gaussian integral. ) Applying Equations (21) and (22) into Equation (23), we find the thermal and active components of MSD scale as

(25) $$\begin{array}{c} {\langle \delta x{\left(t\right)}^{2}\rangle }_{th}≃\frac{k_{B}T}{N\gamma }{\left(\frac{\gamma }{k}\right)}^{1/2}t^{1/2}, \end{array}$$

(26) $$\begin{array}{c} {\langle \delta x{\left(t\right)}^{2}\rangle }_{A}≃\left\{\begin{array}{cc} \frac{A}{N\gamma^{2}}{\left(\frac{\gamma }{k}\right)}^{1/2}t^{3/2}, & (t\ll \tau_{A}),\\ \frac{A\tau_{A}}{N\gamma^{2}}{\left(\frac{\gamma }{k}\right)}^{1/2}t^{1/2}, & (t\gg \tau_{A}). \end{array}\right. \end{array}$$

**Figure 3:**
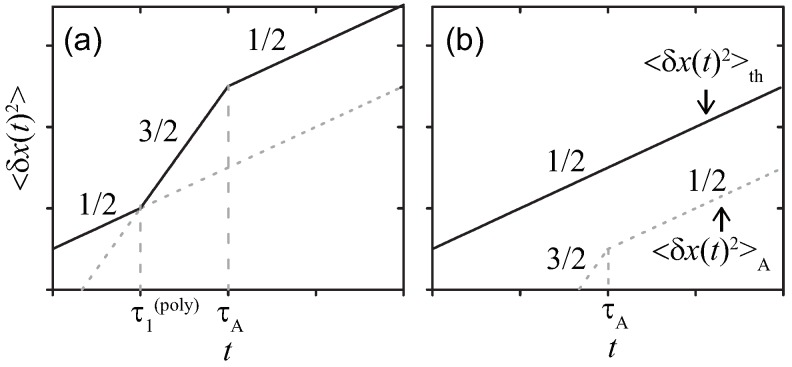
Sketches of MSDs $\langle \delta x{\left(t\right)}^{2}\rangle$ for the Rouse polymer in a viscous media at double-logarithmic scales. The active noise with (**a**) strong $\gamma k_{B}T/\tau_{A}<A$ or (**b**) weak $\gamma k_{B}T/\tau_{A}>A$ magnitude, respectively.

The net results on $\langle \delta x{\left(t\right)}^{2}\rangle ={\langle \delta x{\left(t\right)}^{2}\rangle }_{th}+{\langle \delta x{\left(t\right)}^{2}\rangle }_{A}$ are summarized in Figure 3, which is coincident with those found in Refs. [12,13] ( See Ref. [13], which discusses the realistic modifications in detail by taking account of the finite extensibility). The characteristic time $\tau_{1}^{\left(poly\right)}=\gamma k_{B}T/A$ is introduced for the polymer and obtained by comparing ${\langle \delta x{\left(t\right)}^{2}\rangle }_{A}$ with ${\langle \delta x{\left(t\right)}^{2}\rangle }_{th}$ similarly to $\tau_{1}$ for the simple probe. Note that $\tau_{2}^{\left(poly\right)}$ being the counterpart to $\tau_{2}$ is not observed for the polymer, i.e., the effect of the active noise lasts after dominating.

#### 3.1.2. Diverse Polymers

Section 3.1.2 generalizes the results of the position fluctuation for the Rouse model in the viscous media to take into account various factors, which may become relevant in certain situations. First, we introduce the Flory exponent ν, which associates the polymer spatial size $R_{M}$ with the number of monomers (or mass) *M* that constitutes the polymer as $R_{M}∼M^{\nu }$ [14,18,19]. Besides ν=1/2 for the ideal polymer, for example, if the self-avoidance is effective as the repulsions between monomers, the polymer swells as ν≃0.588 in three dimensions or ν=3/4 in two dimensions. Another example with non-ideal conformation is a fractal crumpled globule with ν=1/3. Next, the monomer friction/mobility property can be altered from a simple free-draining mechanism in the Rouse model, e.g., to the non-draining one, where the medium flow induced by a monomer motion affects the motion of the distant ones [14,18,19]. In our formalism, such an effect is treated by introducing the exponent *z*, which describes how the drag coefficient $\Gamma_{R}$ of the chain depends on its spatial size *R*, i.e., $\Gamma_{R}∼R^{z-2}$ such that the longest relaxation time is reproduced to scale as $∼R^{z}$ if in the viscous fluid. The last feature is the branchings, which may be characterized by the spectral dimension $d_{s}$, relating the number of the monomers *M* to the linear connecting size *N* as $M≃N^{d_{s}}$ [22,23]. While the linear polymer has $d_{s}=1$, the polymerized membrane does $d_{s}=2$ [24,25]. The polymer being randomly branched, it sits at $d_{s}≃4/3$ between them [23]. We can incorporate those properties by replacing the coefficients of Equations (20) with:

(27) $$\begin{array}{c} \gamma_{q}=\gamma {\left(\frac{q}{N}\right)}^{d_{s}-\left(z-2\right)\nu d_{s}},\;k_{q}=k{\left(\frac{q}{N}\right)}^{d_{s}+2\nu d_{s}} \end{array}$$

and by modifying the transformation formula in accordance with the density of states in *q*-space

(28) $$\begin{array}{ccc} h_{q,n} & = & \frac{1}{N}{\left(\frac{q}{N}\right)}^{d_{s}-1}\cos\left(\frac{\pi qn}{N}\right),\\ h_{q,n}^{†} & = & 2{\left(\frac{q}{N}\right)}^{d_{s}-1}\cos\left(\frac{\pi qn}{N}\right). \end{array}$$

Note that, if the coefficients are expressed with the number of monomers $m∼{\left(N/q\right)}^{d_{s}}$, the spectral dimension $d_{s}$ does not explicitly appear as $\gamma_{q}∼m^{-1+(z-2)\nu }$ and $k_{q}∼m^{-1-2\nu }$, because $d_{s}$ is related to the spectrum of density of sates in *q*-space.

In addition, although the temporal component Equation (22) is not modified, the wavenumber dependency is embedded into the noise correlations:

(29) $$\begin{array}{c} \langle \Xi_{q}^{\left(x\right)}\left(t\right)\Xi_{q^{\prime }}^{\left(x\right)}\left(t^{\prime }\right)\rangle =\frac{\gamma_{q}}{N^{d_{s}}\gamma }{\left(\frac{q}{N}\right)}^{1-d_{s}}\delta_{qq^{\prime }}\Phi \left(t-t^{\prime }\right). \end{array}$$

To obtain the MSD, we follow the same line as that in Equation (23)

(30) $$\begin{array}{cc} & \langle \delta x{\left(t\right)}^{2}\rangle \\ ≃ & \int_{0}^{t}ds\int_{0}^{t}ds^{\prime }\;\frac{1}{N^{d_{s}}\gamma^{2}}{\left(\frac{\gamma }{k}\right)}^{1-2/z}{\left|2t-s-s^{\prime }\right|}^{2/z-1}\Phi \left(s-s^{\prime }\right). \end{array}$$

We then arrive at the general results for the polymers in the viscous media:

(31) $$\begin{array}{c} {\langle \delta x{\left(t\right)}^{2}\rangle }_{th}≃\frac{k_{B}T}{N^{d_{s}}\gamma }{\left(\frac{\gamma }{k}\right)}^{1-2/z}t^{2/z}, \end{array}$$

(32) $$\begin{array}{c} {\langle \delta x{\left(t\right)}^{2}\rangle }_{A}≃\left\{\begin{array}{cc} \frac{A}{N^{d_{s}}\gamma^{2}}{\left(\frac{\gamma }{k}\right)}^{1-2/z}t^{1+2/z}, & (t\ll \tau_{A}),\\ \frac{A\tau_{A}}{N^{d_{s}}\gamma^{2}}{\left(\frac{\gamma }{k}\right)}^{1-2/z}t^{2/z}, & (t\gg \tau_{A}). \end{array}\right. \end{array}$$

It is noticeable that ν, $d_{s}$ are not seen in the indices of the power law for *t*, but only the index *z* explicitly survives in the last results. Equations (31) and (32) are more generalized, and, indeed, the result on a simple linear chain Rouse model in Section 3.1.1 is recovered in a special case of the free-draining $z=2+\nu^{-1}$ and the ideal chain statistics ν=1/2 with $d_{s}=1$. In addition, although Equations (31) and (32) include the other polymers, the same characteristic time $\tau_{1}^{\left(poly\right)}=\gamma k_{B}T/A$ as that for the Rouse is adopted.

### 3.2. Momentum Transfer Fluctuations

This part considers the fluctuations of $p\left(t\right)=\int_{0}^{t}ds\;f\left(t\right)$ in the paired observation, where the force f(t) acting on the tagged monomer is measured while the tagged monomer’s position is fixed at x(t)=x(0) or moved as x(t)=vt+x(0) with *v* being constant. The corresponding experimental systems are feasible, e.g., by using the trap optical tweezers like Ref. [32]. Note that, if the tagged monomer is moved, the variances $\langle {\left(\delta p\left(t\right)-\langle \delta p(t)\rangle \right)}^{2}\rangle$ should be measured [26,27] instead of the MSDs $\langle \delta p{\left(t\right)}^{2}\rangle$. We construct the discussion based on the normal modes here too. The conversion of the variables from and to the normal modes are carried out in the same way as Equation (17) with Equation (28), e.g., $F_{q}\left(t\right)=\int_{0}^{N}dn\;f_{n}\left(t\right)h_{q,n}$. Note that $f_{n}\left(t\right)$ denotes the force magnitude including the applied external and the random forces. As derived in Ref. [26], the time evolutions for the observable $F_{q}\left(t\right)$ are written by

(33) $$\begin{array}{c} m_{q}\frac{dF_{q}\left(t\right)}{dt}=-g_{q}F_{q}\left(t\right)+V_{q}\left(t\right)+\Xi_{q}^{\left(p\right)}\left(t\right), \end{array}$$

where the coefficients are given as

(34) $$\begin{array}{ccc} g_{q} & = & \frac{1}{\gamma_{q}}{\left(\frac{q}{N}\right)}^{2d_{s}}=\frac{1}{\gamma }{\left(\frac{q}{N}\right)}^{d_{s}+\left(z-2\right)\nu d_{s}}, \end{array}$$

(35) $$\begin{array}{ccc} m_{q} & = & \frac{1}{k_{q}}{\left(\frac{q}{N}\right)}^{2d_{s}}=\frac{1}{k}{\left(\frac{q}{N}\right)}^{d_{s}-2\nu d_{s}}. \end{array}$$

In Equation (33), $V_{q}\left(t\right)$ denotes the controlled velocity, and $\Xi_{q}^{\left(p\right)}\left(t\right)$ is the zero mean noise, i.e., the velocity fluctuation arising from the thermal and the active random forces. The noise correlation is converted as

(36) $$\begin{array}{c} \langle \Xi_{q}^{\left(p\right)}\left(t\right)\Xi_{q^{\prime }}^{\left(p\right)}\left(t^{\prime }\right)\rangle =\frac{g_{q}}{N^{d_{s}}\gamma }{\left(\frac{q}{N}\right)}^{1-d_{s}}\delta_{qq^{\prime }}\Phi \left(t-t^{\prime }\right) \end{array}$$

with the same temporal scaling as Φ(t) in Equation (29). Note that, throughout the paper, the Lengevin descriptions like Equations (15), (19) and (33) adopt the overdamped time scale, where the position is appropriate as the observable. In the protocol to monitor the momentum transfer, we regard the controlled velocity as the results of the controlled position.

The derivation of Equation (33) is discussed in Ref. [26] in detail from the consideration of the kernel conversions of the GLE. Instead here, let us give a brief interpretation of Equation (33). If the polymer is free ($V_{q}\left(t\right)=0$), the mean applied force is always zero $\langle F_{q}\left(t\right)\rangle =0$, around which $F_{q}\left(t\right)$ fluctuates. On the other hand, if $n_{0}$th monomer is temporally controlled as $x_{n_{0}}\left(t\right)=vt+x_{n_{0}}\left(0\right)$ for t>0, the mean applied force shifts from $\langle F_{q}\left(t\right)\rangle =0$ to $vh_{q,n_{0}}^{†}/g_{q}$ in the relaxation time $\tau_{q}=m_{q}/g_{q}$ with the fluctuations around the mean. This mean transient process is traced by the velocity balance $m_{q}d\langle F_{q}\left(t\right)\rangle /dt=-g_{q}\langle F_{q}\left(t\right)\rangle$, which represents that the polymer relaxes owing to the entropic elasticity. In addition, eliminating the bracket from the mean velocity balance, and adding $V_{q}\left(t\right)$ and $\Xi_{q}^{\left(p\right)}\left(t\right)$, we arrive at Equation (33).

Based on Equation (33), we get the scaling of $\langle \delta p{\left(t\right)}^{2}\rangle$. Calculating $f_{n}\left(t\right)=\sum_{q}F_{q}\left(t\right)h_{q,n}^{†}$ with Equations (33)–(35), we obtain the force correlation as

(37) $$\begin{array}{ccc} \langle \delta f\left(t\right)\delta f\left(t^{\prime }\right)\rangle & ≃ & \int_{0}^{t}ds\int_{0}^{t^{\prime }}ds^{\prime }\;\frac{1}{N^{d_{s}}}{\left(\frac{\gamma }{k}\right)}^{2/z-1}\\ & & \times |t-s+t^{\prime }-s^{\prime }{|}^{-1-2/z}\Phi \left(s-s^{\prime }\right). \end{array}$$

Applying Equation (22) into Equation (37), and taking account of the double time integrals, we obtain the MSD scaling:

(38) $$\begin{array}{c} {\langle \delta p{\left(t\right)}^{2}\rangle }_{th}≃\frac{\gamma k_{B}T}{N^{d_{s}}}{\left(\frac{\gamma }{k}\right)}^{2/z-1}t^{2-2/z}, \end{array}$$

(39) $$\begin{array}{c} {\langle \delta p{\left(t\right)}^{2}\rangle }_{A}≃\left\{\begin{array}{cc} \frac{A}{N^{d_{s}}}{\left(\frac{\gamma }{k}\right)}^{2/z-1}t^{3-2/z}, & (t\ll \tau_{A}),\\ \frac{A\tau_{A}}{N^{d_{s}}}{\left(\frac{\gamma }{k}\right)}^{2/z-1}t^{2-2/z}, & (t\gg \tau_{A}). \end{array}\right. \end{array}$$

Combining Equations (31), (32), (38) and (39), the results corresponding to Equation (3) are replaced by

(40) $$\begin{array}{c} \alpha^{\left(x\right)}+\alpha^{\left(p\right)}-2=\left\{\begin{array}{cc} 0, & (\mathrm{thermal}),\\ \left\{\begin{array}{cc} 2 & (t\ll \tau_{A}),\\ 0 & (t\gg \tau_{A}), \end{array}\right. & (\mathrm{active}). \end{array}\right. \end{array}$$

Equation (40) for $t\ll \tau_{A}$ exhibits the qualitative deviations from Equation (3). On the other hand, the same relation as Equation (3) is found for $t\gg \tau_{A}$ since the noise correlations get lost on this time window, and the noise may be treated as the delta-function correlation; thus, the temporal property of the noise is the same as the thermal noise with the temperature replaced by the effective one at the coarse-grained time scaling $t\gg \tau_{A}$.

**Figure 4:**
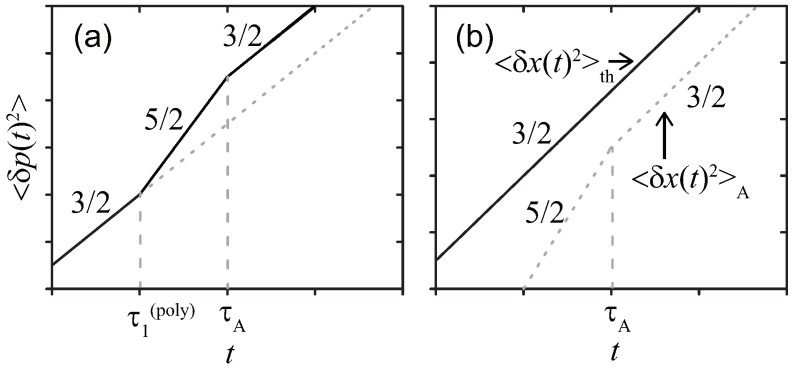
Sketches of MSDs $\langle \delta p{\left(t\right)}^{2}\rangle$ for the Rouse polymer in a viscous media at double-logarithmic scales. The active noise with (**a**) strong $\gamma k_{B}T/\tau_{A}<A$ or (**b**) weak $\gamma k_{B}T/\tau_{A}>A$ magnitude, respectively.

Figure 4 summarizes the MSDs of p(t) for the Rouse polymer. As in Section 3.1.2, even for the other polymers with different *z*, the regime shifts with the characteristic time $\tau_{1}^{\left(poly\right)}=\gamma k_{B}T/A$ are not qualitatively altered.

## 4. Discussion

As possible generalization of our analysis, we discuss the effect of medium viscoelasticity and the power–law correlated active noise.

### 4.1. Viscoelastic Media

In standard models of polymer dynamics, each monomer is assumed to experience a viscous drag force upon its movement, which corresponds to an instantaneous response to the velocity. As a possible extension of this, one can, at least, imagine a model, in which the monomer motion is accompanied by a viscoelastic resistance, evolving with the movement memory. There are some recent claims that such a polymer model embedded in a viscoelastic medium may account for the motion of chromosomal loci in cells [8,11,12,13]. We have carried out analysis for such a model and found that the same relations as Equation (40) are maintained, i.e., we find the same deviation from equilibrium relation (Equation (3)) for $t\ll \tau_{A}$ and the recovery for $t\gg \tau_{A}$ (see Appendix A for details).

### 4.2. Power-Law Correlated Active Noises

In literature, the active noise is often supposed to have the exponential temporal correlation. Here, we consider the conceivable possibility that the active noise has the power–law correlation for $t\ll \tau_{A}$. While most elements in our analysis so far are intact, the temporal evolution of the active noise correlation is replaced with

(41) $$\begin{array}{c} \Phi_{A}\left(t-t^{\prime }\right)≃A_{\psi }{\left|t-t^{\prime }\right|}^{-\psi }e^{-|t-t^{\prime }|/\tau_{A}}, \end{array}$$

where $\Phi_{A}\left(\cdot \right)$ indicates the active component of the noise correlation. This power–law active noise alters the MSD exponents as:

(42) $$\begin{array}{c} {\langle \delta x{\left(t\right)}^{2}\rangle }_{A}∼\left\{\begin{array}{cc} t^{2\lambda \times (2/z)-\psi }, & (t\ll \tau_{A}),\\ t^{\left(2\lambda -1)\times (2/z\right)}, & (t\gg \tau_{A}), \end{array}\right. \end{array}$$

(43) $$\begin{array}{c} {\langle \delta p{\left(t\right)}^{2}\rangle }_{A}∼\left\{\begin{array}{cc} t^{4-2\lambda \times (2/z)-\psi }, & (t\ll \tau_{A}),\\ t^{2-(2\lambda -1)\times (2/z)}, & (t\gg \tau_{A}). \end{array}\right. \end{array}$$

It is noticeable that the additional factors $t^{-\psi }$ appear only for $t\ll \tau_{A}$ in both the cases. Taking the sum of the exponents, the inserted −ψ is not canceled out, but emerges as:

(44) $$\begin{array}{ccc} \alpha^{\left(x\right)}+\alpha^{\left(p\right)}-2 & = & \left\{\begin{array}{cc} 2-2\psi , & (t\ll \tau_{A}),\\ 0, & (t\gg \tau_{A}). \end{array}\right. \end{array}$$

The MSD exponents are expressed by the combination of λ, *z*, and ψ as seen in Equations (42) and (43), and thus it would impose on us the hard task to determine ψ from either of $\alpha^{\left(x\right)}$ or $\alpha^{\left(p\right)}$. However, the sum of both $\alpha^{\left(x\right)}$ and $\alpha^{\left(p\right)}$ allows us to extract ψ. Thus, Equation (44) would be used as the indicator to specify the property of the noise.

## 5. Conclusions

We have investigated the paired anomalous diffusions, one measured by the tracer’s position fluctuation in force-free protocol, and the other measured by the force fluctuation acting on the probe, which is fixed in space, driven by thermal and active noises. We have shown that, while quite generally the sum of MSD exponents in these paired protocols obeys relation (3) in thermal equilibrium, the deviation from such an equilibrium relation may occur in systems driven by active noise. We should note that the MSDs do not always strictly follow power laws in complex environment, since various crossover effects may hinder the clear identification of the power law slope, and there may be other physical mechanisms [33,34] such as caging effects for anomalous diffusion. Nonetheless, we hope that proposed way of analysis, i.e., combining both the paired observations of the anomalous diffusion may be useful to extract the physical properties of the system, where several different factors are at work simultaneously to generate complex dynamical behaviors, e.g., in cells and cellular nuclei.

## Appendix A. Polymer in Viscoelastic Media

There are some suggestions in literature that the dynamics of chromosomal loci in living cells may be described by a model, in which the motion of monomers is influenced by the medium viscoelastic effect. Although it is not clear at present if the medium viscoelasticity is really a relevant factor in the scale of monomers, it may be interesting to see the prediction of such a model. A so-called viscoelastic Rouse model was introduced and analyzed in Ref. [8]. The effect of active noise on the viscoelastic Rouse model was studied in Ref. [12]. In Ref. [13], we generalized these studies to encompass the effect associated with non-ideal conformations, i.e., swelling due to self-avoidance, fractal crumpled conformation, etc., and constructed a scaling theory for active and anomalous diffusion of chromosomal loci (see Ref. [11] for a similar work). Here, we aim to reproduce our previous results obtained in Ref. [13] in terms of an approximate mode analysis. We then make use of the mode analysis technique to investigate the fluctuation of the momentum transfer in the position-fixed protocol.

### Appendix A.1. Position Fluctuations

We shall first recall the scaling argument in Ref. [13]. In the following calculation, it is useful to introduce an auxiliary index ζ in such a way that the longest relaxation time of *N*-(linear) strand is

(A1) $$\begin{array}{c} \tau^{\lambda }∼N^{\zeta }, \end{array}$$

where λ is the stress relaxation exponent of the viscoelastic medium (see Section 2). In the scheme of the mode analysis approximation, this amounts to considering the following equation of motion for monomer beads

(A2) $$\begin{array}{c} D_{t}^{\lambda }x_{n}\left(t\right)=D_{n}^{\zeta }x_{n}\left(t\right), \end{array}$$

where the noise term is omitted and the operator $D_{t}^{\lambda }$ or $D_{n}^{\zeta }$ represents the fractional derivative with respect to time or “space” (that is the coordinate along the chain), respectively [13]. While the order λ of time derivative coincides with the stress relaxation exponent of the medium, the fractional order ζ of spacial derivative is associated with the long-range interaction between distant monomers along the connectivity [14,35], and should be determined in a self-consistent way from physical requirement. To achieve this task of self-consistency, in Ref. [13], we obtained two different expressions for the MSD exponent $\alpha^{\left(x\right)}$

(A3) $$\begin{array}{c} \alpha^{\left(x\right)}=\alpha_{SP}^{\left(x\right)}-\lambda \left(z-2\right)\nu d_{s}/\zeta \end{array}$$

and

(A4) $$\begin{array}{c} \alpha^{\left(x\right)}=2\lambda \nu d_{s}/\zeta , \end{array}$$

based on physically independent arguments, where $\alpha_{SP}^{\left(x\right)}$ is the MSD exponent for the simple probe analyzed in Section 2, i.e., for thermal component $\alpha_{SP}^{\left(x\right)}=\lambda$ (Equation (8)), and for active component, $\alpha_{SP}^{\left(x\right)}=2\lambda$ ($\tau \ll \tau_{A}$) and $\alpha_{SP}^{\left(x\right)}=2\lambda -1$ ($\tau \gg \tau_{A}$) (Equation (9)). Then, requiring the consistency between these arguments, we found

(A5) $$\begin{array}{c} \zeta =\left\{\begin{array}{cc} d_{s}\nu_{eq}z_{eq}, & (\mathrm{thermal}),\\ \left\{\begin{array}{cc} d_{s}\nu_{A}z_{A}/2, & (t\ll \tau_{A}),\\ d_{s}\nu_{A}z_{A}\lambda /\left(2\lambda -1\right), & (t\gg \tau_{A}), \end{array}\right. & (\mathrm{active}), \end{array}\right. \end{array}$$

where we distinguish exponents ν and *z* in active component from those in thermal components, as the active noise may affect the chain conformation, thus modifying the value of ν from that in equilibrium case. Observe that in the special case of a linear polymer $d_{s}=1$ in thermal system with ideal chain statistics $v_{eq}=1/2$ and free-draining dynamics $z_{eq}=2+\nu_{eq}^{-1}=4$, we recover a standard Rouse model exponent ζ=2. However, the long-range interaction, either dynamic (cf. hydrodynamic interaction) or static (cf. non-ideal conformation with ν≠1/2) results in a fractional value of ζ. Plugging Equation (A5) into Equation (A3) or Equation (A4), we found $\langle \delta x{\left(t\right)}^{2}\rangle ={\langle \delta x{\left(t\right)}^{2}\rangle }_{th}+{\langle \delta x{\left(t\right)}^{2}\rangle }_{A}$ with

(A6) $$\begin{array}{c} {\langle \delta x{\left(t\right)}^{2}\rangle }_{th}∼t^{\lambda \times (2/z_{eq})}, \end{array}$$

(A7) $$\begin{array}{c} {\langle \delta x{\left(t\right)}^{2}\rangle }_{A},∼\left\{\begin{array}{cc} t^{2\lambda \times (2/z_{A})}, & (t\ll \tau_{A}),\\ t^{\left(2\lambda -1\right)\times \left(2/z_{A}\right)}, & (t\gg \tau_{A}). \end{array}\right. \end{array}$$

We now move on the mode analysis to redrive Equation (A3). In the mode space, the equation of motion in the viscoelastic media is rewritten as

(A8) $$\begin{array}{c} \int_{0}^{t}ds\;\gamma_{q}G\left(t-s\right)\frac{dX_{q}\left(s\right)}{ds}=-k_{q}X_{q}\left(t\right)+F_{q}\left(t\right)+\Xi_{q}^{\left(x\right)}\left(t\right), \end{array}$$

where $G\left(t\right)\equiv \left(\Gamma_{\lambda }/\gamma \right)\;t^{-\lambda }$. Note that An additional factor $\gamma^{-1}$ compared to the definition in Equation (6) in Section 2 is introduced just to adjust the dimension of $\gamma_{q}$ in Equation (A9). The wavenumber-dependent coefficients are replaced by

(A9) $$\begin{array}{c} \gamma_{q}=\gamma {\left(\frac{q}{N}\right)}^{d_{s}-\left(z-2\right)\nu d_{s}},\;k_{q}=k{\left(\frac{q}{N}\right)}^{d_{s}+\zeta -\left(z-2\right)\nu d_{s}}. \end{array}$$

whose indices are assigned according to our definition. First, in order to meet with Equation (A1), the ratio of the left to the right Equation (A9) is set as $\tau_{q}^{\lambda }\equiv \left(\gamma_{q}/k_{q}\right)=\left(\gamma /k\right){\left(N/q\right)}^{\zeta }$ with $\tau_{q}$ being *q*-th mode’s relaxation time. In addition, our definition of *z* (see the beginning of Section 3.1.2) determines the index of $\gamma_{q}$ to be independent of the other indices relevant to the dynamics like ζ. Bear in mind that, comparing Equations (A9) with Equations (27), we find the difference only in $k_{q}$.

The temporal components in the noise correlations ($\Phi (t-t^{\prime })$ in Equation (29)) are also replaced by

(A10) $$\begin{array}{c} \Phi^{\left(x\right)}\left(t-t^{\prime }\right)=k_{B}T\gamma G\left(t-t^{\prime }\right)+Ae^{-|t-t^{\prime }|/\tau_{A}}. \end{array}$$

Note that *q*-dependency in the noise correlation is unchanged from Equation (29). Those replacements by Equations (A9) and (A10) modify the calculation outline of the MSD (see Appendix B for detail):

(A11) $$\begin{array}{cc} & \langle \delta x{\left(t\right)}^{2}\rangle \\ ∼ & \int_{0}^{t}ds\int_{0}^{t}ds^{\prime }\int_{0}^{s}du\int_{0}^{s^{\prime }}du^{\prime }\;\sum_{q,q^{\prime }}{\left(s-u\right)}^{\lambda -2}{\left(s^{\prime }-u^{\prime }\right)}^{\lambda -2}\\ & \times \frac{\langle \Xi_{q}^{\left(x\right)}\left(u\right)\Xi_{q^{\prime }}^{\left(x\right)}\left(u^{\prime }\right)\rangle }{\gamma_{q}\gamma_{q^{\prime }}}\\ & \times E_{\lambda ,1}\left(-\frac{{\left(t-s\right)}^{\lambda }}{\left(\gamma_{q}/k_{q}\right)\tau_{u}^{\lambda -1}}\right)E_{\lambda ,1}\left(-\frac{{\left(t-s^{\prime }\right)}^{\lambda }}{\left(\gamma_{q^{\prime }}/k_{q^{\prime }}\right)\tau_{u}^{\lambda -1}}\right)\\ ∼ & \int_{0}^{t}ds\int_{0}^{t}ds^{\prime }\int_{0}^{s}du\int_{0}^{s^{\prime }}du^{\prime }\;{\left(s-u\right)}^{\lambda -2}{\left(s^{\prime }-u^{\prime }\right)}^{\lambda -2}\\ & \times \Phi^{\left(x\right)}\left(u-u^{\prime }\right){\left[{\left(t-s\right)}^{\lambda }+{\left(t-s^{\prime }\right)}^{\lambda }\right]}^{-\left(z-2\right)\nu d_{s}/\zeta }, \end{array}$$

where $\tau_{u}\equiv \gamma /k$ is the characteristic relaxation time at the monomer scale, and $E_{\alpha ,\beta }\left(\cdot \right)$ denotes the Mittag–Leffler function. To reach the final expression, we approximate the Mittag–Leffler function with its short time asymptotic behavior $E_{\alpha ,1}\left(-{\left(t/T\right)}^{\alpha }\right)∼\exp\left[-{\left(t/T\right)}^{\alpha }\right]$ for t≪T, which dominates the scaling behavior. Evaluating the time integral in Equation (A11), we obtain the MSD scaling in the form of Equation (A3).

### Appendix A.2. Momentum Fluctuations

Next, let us find the fluctuations of the other variable p(t). On the mode space, the time evolution of $F_{q}\left(t\right)$ in the viscoelastic media is modified by

(A12) $$\begin{array}{c} m_{q}\frac{dF_{q}\left(s\right)}{ds}=-\int_{0}^{t}ds\;g_{q}M\left(t-s\right)F_{q}\left(s\right)+V_{q}\left(t\right)+\Xi_{q}^{\left(p\right)}\left(t\right), \end{array}$$

where the kernel $M\left(t\right)\equiv \left[\gamma^{3}\left(1-\lambda \right)\sin\pi (1-\lambda )/\left(\pi \Gamma_{\lambda }k^{2}\right)\right]t^{\lambda -2}$ satisfies $\hat{G}\left(\omega \right)\hat{M}\left(\omega \right)=1$ on the Laplace domain. Using ζ, the wavenumber-dependent coefficients are altered as

(A13) $$\begin{array}{c} g_{q}=\frac{1}{\gamma }{\left(\frac{q}{N}\right)}^{d_{s}+\left(z-2\right)\nu d_{s}},\;m_{q}=\frac{1}{k}{\left(\frac{q}{N}\right)}^{d_{s}-\zeta +\left(z-2\right)\nu d_{s}}. \end{array}$$

The noise correlation is represented as Equation (36) with its temporal part given by

(A14) $$\begin{array}{c} \Phi^{\left(p\right)}\left(t-t^{\prime }\right)=-\gamma k_{B}TM\left(t-t^{\prime }\right)+Ae^{-|t-t^{\prime }|/\tau_{A}}. \end{array}$$

Note that the thermal component of the noise correlation evolves in a different manner from Equation (A10). Like Equation (A11), the asymptotic behaviors of the force correlation is obtained as

(A15) $$\begin{array}{cc} & \langle \delta f\left(t\right)\delta f\left(t^{\prime }\right)\rangle \\ ∼ & \int_{0}^{t}ds\int_{0}^{t^{\prime }}ds^{\prime }\;{\left|{\left(t-s\right)}^{\lambda }+{\left(t^{\prime }-s^{\prime }\right)}^{\lambda }\right|}^{-2+\left(z-2\right)\nu d_{s}/\zeta }\\ & \times \Phi^{\left(p\right)}\left(s-s^{\prime }\right). \end{array}$$

As in the calculation from Equation (37) to Equation (39), the scalings are estimated as

(A16) $$\begin{array}{c} {\langle \delta p{\left(t\right)}^{2}\rangle }_{th}∼t^{2-\lambda +\lambda \left(z-2\right)\nu d_{s}/\zeta }, \end{array}$$

(A17) $$\begin{array}{c} {\langle \delta p{\left(t\right)}^{2}\rangle }_{A}∼\left\{\begin{array}{cc} t^{4-2\lambda +\lambda \left(z-2\right)\nu d_{s}/\zeta }, & (t\ll \tau_{A}),\\ t^{2-2\lambda +1+\lambda \left(z-2\right)\nu d_{s}/\zeta }, & (t\gg \tau_{A}). \end{array}\right. \end{array}$$

To determine ζ, we adopt Equation (A5) and arrive at

(A18) $$\begin{array}{c} {\langle \delta p{\left(t\right)}^{2}\rangle }_{th}≃t^{2-\lambda \times (2/z_{eq})}, \end{array}$$

(A19) $$\begin{array}{c} {\langle \delta p{\left(t\right)}^{2}\rangle }_{A}∼\left\{\begin{array}{cc} t^{4-2\lambda \times (2/z_{A})}, & (t\ll \tau_{A}),\\ t^{2-\left(2\lambda -1\right)\times \left(2/z_{A}\right)}, & (t\gg \tau_{A}). \end{array}\right. \end{array}$$

The summation of Equations (A6), (A7), (A18) and (A19) gives the indicator for the deviations from Equation (3) as

(A20) $$\begin{array}{ccc} \alpha^{\left(x\right)}+\alpha^{\left(p\right)}-2 & = & \left\{\begin{array}{cc} 0, & (\mathrm{thermal}),\\ \left\{\begin{array}{cc} 2 & (t\ll \tau_{A}),\\ 0 & (t\gg \tau_{A}), \end{array}\right. & (\mathrm{active}). \end{array}\right. \end{array}$$

It is noticeable that, in the active noise with the exponential function correlations, the deviations are found for $t\ll \tau_{A}$ and quantified without any indices like ν or *z*. In addition, as argued in Section 4.2, if the active noise displays the power–law correlations, the same relation Equation (44) holds:

*—active media $t\gg \tau_{A}$—*.

We here identify the anomalous diffusion exponents $\alpha^{\left(x\right)}$ or $\alpha^{\left(p\right)}$ according to the temporal regimes distinguished by $\tau_{A}$. Let us first see $t\gg \tau_{A}$. At the time scale when the active noise correlations get lost, the polymer is regarded as being immersed in equilibrium with the effective temperature, implying that both the asymptotic tension propagation along the chain between in the thermal and in the active noises are identical, i.e., $\zeta_{A}=\zeta_{eq}$ in Equation (A2). Then, to identify $\alpha_{A}^{\left(x\right)}$, we here utilize the condition $\zeta =2\lambda z\nu d_{s}/\alpha_{SP}^{\left(x\right)}$ led by the equivalency of Equations (A3) and (A4), which yields

(A21) $$\begin{array}{ccc} \frac{\alpha_{SP,A}^{\left(x\right)}}{z_{A}\nu_{A}} & = & \frac{\alpha_{SP,eq}^{\left(x\right)}}{z_{eq}\nu_{eq}}. \end{array}$$

Even in the active media, the equilibrium ones for *z* are acknowledged as $z_{A}=3$ for the non-draining or $z_{A}=2+1/\nu_{A}$ for the free-draining, Picking Equation (A21) up and combining it with Equations (A7) and (A19) for $t\gg \tau_{A}$, we find

(A22) $$\begin{array}{c} \alpha^{\left(x\right)}=\left\{\begin{array}{cc} \frac{2}{3}\left(2\lambda -1\right) & (non–draining),\\ 2\lambda -1-\frac{\lambda }{2\nu_{eq}+1} & (free–draining), \end{array}\right. \end{array}$$

(A23) $$\begin{array}{c} \alpha^{\left(p\right)}=\left\{\begin{array}{cc} \frac{2}{3}\left(-2\lambda +4\right) & (non–draining),\\ -2\lambda +3+\frac{\lambda }{2\nu_{eq}+1} & (free–draining). \end{array}\right. \end{array}$$

Therefore, $\alpha^{\left(x\right)}+\alpha^{\left(p\right)}=2$ holds even in the presence of active noise. These MSD exponents coincide with those in equilibrium (Equations (A6) and (A18), respectively) when λ=1, i.e., in a viscous fluid:

*—active media $t\ll \tau_{A}$—-*.

At the short time scale $t\ll \tau_{A}$, the active noise may manifest the specific local characteristics in the polymer. Illustrate the case of a Rouse polymer, which is modeled as a sequence of beads connected by the harmonic potential springs. As in Refs. [12,13], the position of the tagged monomer for $\tau_{u}\ll t\ll \tau_{A}$ undergoes the superdiffsuion $\langle \delta x{\left(t\right)}^{2}\rangle ∼t^{3/2}$ (see Figure 3a), and the polymer gets overswollen with $\nu_{A}>1$. Those features might be pathologic as discussed in Ref. [13]. As one of the realistic conditions, we next see how $\alpha^{\left(x\right)}$ and $\alpha^{\left(p\right)}$ are altered for the finitely extensible polymer. The finite extension prohibits the overswelling by imposing the upper bound $\nu_{A}=1$, and thus we have $\alpha^{\left(x\right)}=4\lambda /3$ with Equation (A7) and $z_{A}=2+1/\nu_{A}$ [13]. In addition, because the relevant polymer indices are assumed not to change in the same active noise, we can use Equation (A20) to get $\alpha^{\left(p\right)}$ for $t\ll \tau_{A}$, and arrive at $\alpha^{\left(p\right)}=4-4\lambda /3$. This always displays the overballistic manner for the practical condition (0<λ<1). Finally, we summarize MSD scalings of a tagged monomer in this time regime (see the first line of Equations (A7) and (A19)):

(A24) $$\begin{array}{c} \alpha^{\left(x\right)}=\left\{\begin{array}{cc} \frac{4\lambda }{3} & (non–draining),\\ \frac{4\lambda }{2+1/\nu_{A}} & (free–draining), \end{array}\right. \end{array}$$

(A25) $$\begin{array}{c} \alpha^{\left(p\right)}=\left\{\begin{array}{cc} 4-\frac{4\lambda }{3} & (non–draining),\\ 4-\frac{4\lambda }{2+1/\nu_{A}} & (free–draining). \end{array}\right. \end{array}$$

It is noticeable that the finite extensible polymer ($\nu_{A}=1$) does not distinguish non- or free-draining in Equations (A24) and (A25).

## Appendix B. Mode Analyses

This part gives the calculations for the viscoelastic media in detail.

On the Laplace domain, Equation (A8) of motion is written as

(A26) $$\begin{array}{c} \gamma_{q}\hat{G}\left(\omega \right)\left[\omega {\hat{X}}_{q}\left(\omega \right)-X_{q}\left(0\right)\right]=-k_{q}{\hat{X}}_{q}\left(\omega \right)+{\hat{F}}_{q}\left(\omega \right)+{\hat{\Xi }}_{q}^{\left(x\right)}\left(\omega \right), \end{array}$$

where $\hat{G}\left(\omega \right)≃\omega^{\lambda -1}$. The solution to Equation (A26) is given by

(A27) $$\begin{array}{c} {\hat{X}}_{q}\left(\omega \right)=\frac{\hat{G}{\left(\omega \right)}^{-1}}{\gamma_{q}}\frac{\left[{\hat{F}}_{q}\left(\omega \right)+{\hat{\Xi }}_{q}^{\left(x\right)}\left(\omega \right)\right]}{\omega +\hat{G}{\left(\omega \right)}^{-1}k_{q}/\gamma_{q}}+\frac{X_{q}\left(0\right)}{\omega +\hat{G}{\left(\omega \right)}^{-1}k_{q}/\gamma_{q}}. \end{array}$$

To inverse Laplace transform Equation (A27), we utilize the Mittag-Leffler function [12,33,36] defined as

(A28) $$\begin{array}{c} E_{\alpha ,\beta }\left(z\right)=\sum_{k=0}^{\infty }\frac{z^{k}}{\Gamma (\alpha k+\beta )}, \end{array}$$

with Γ(·) denoting the Gamma function. At β=1, it is known to satisfy

(A29) $$\begin{array}{c} E_{\alpha ,1}\left(-{\left(\frac{t}{T}\right)}^{\alpha }\right)=L^{-1}\left(\frac{1}{\omega +T^{-\alpha }\omega^{1-\alpha }}\right), \end{array}$$

where $L^{-1}$ denotes the inverse Laplace transform. In the analysis, we use the asymptotic behaviors [33] of

(A30) $$\begin{array}{c} E_{\alpha ,1}\left(-{\left(t/T\right)}^{\alpha }\right)∼\left\{\begin{array}{cc} \exp\left[-{\left(t/T\right)}^{\alpha }/\Gamma \left(1+\alpha \right)\right], & (t\ll T),\\ {\left(t/T\right)}^{-\alpha }/\Gamma \left(1-\alpha \right), & (t\gg T), \end{array}\right. \end{array}$$

Inverse Laplace transforming uation (A27) and superimposing those with respect to *q*, we arrive at

(A31) $$\begin{array}{ccc} x(t) & = & \sum_{q}\int_{0}^{t}ds\int_{0}^{s}du\;{\left(s-u\right)}^{\lambda -2}\tau_{u}^{-(\lambda -1)}\frac{F_{q}\left(u\right)+\Xi_{q}\left(u\right)}{\gamma_{q}}\\ & & \times E_{\lambda ,1}\left(-\frac{{\left(t-s\right)}^{\lambda }}{\left(\gamma_{q}/k_{q}\right)\tau_{u}^{\lambda -1}}\right)h_{q,n}^{†}\\ & & -\sum_{q}X_{q}\left(0\right)\left[1-E_{\lambda ,1}\left(-\frac{t^{\lambda }}{\left(\gamma_{q}/k_{q}\right)\tau_{u}^{\lambda -1}}\right)\right]h_{q,n}^{†}. \end{array}$$

Employing this and putting $F_{q}\left(t\right)=0$, we get the expression of the MSD:

(A32) $$\begin{array}{cc} & \langle \delta x{\left(t\right)}^{2}\rangle \\ = & \int_{0}^{t}ds\int_{0}^{t}ds^{\prime }\int_{0}^{s}du\int_{0}^{s^{\prime }}du^{\prime }\;{\left(s-u\right)}^{\lambda -2}{\left(s^{\prime }-u^{\prime }\right)}^{\lambda -2}r\\ & \times \sum_{q,q^{\prime }}\frac{\langle \Xi_{q}^{\left(x\right)}\left(s\right)\Xi_{q^{\prime }}^{\left(x\right)}\left(s^{\prime }\right)\rangle }{\gamma_{q}\gamma_{q^{\prime }}}\\ & \times E_{\lambda ,1}\left(-\frac{{\left(t-s\right)}^{\lambda }}{\left(\gamma_{q}/k_{q}\right)\tau_{u}^{\lambda -1}}\right)E_{\lambda ,1}\left(-\frac{{\left(t-s^{\prime }\right)}^{\lambda }}{\left(\gamma_{q^{\prime }}/k_{q^{\prime }}\right)\tau_{u}^{\lambda -1}}\right)h_{q,n}^{†}h_{q^{\prime },n}^{†}\\ & +\sum_{q,q^{\prime }}\langle X_{q}\left(0\right)X_{q^{\prime }}\left(0\right)\rangle \left[1-E_{\lambda ,1}\left(-\frac{t^{\lambda }}{\left(\gamma_{q}/k_{q}\right)\tau_{u}^{\lambda -1}}\right)\right]\\ & \times \left[1-E_{\lambda ,1}\left(-\frac{t^{\lambda }}{\left(\gamma_{q^{\prime }}/k_{q^{\prime }}\right)\tau_{u}^{\lambda -1}}\right)\right]h_{q,n}^{†}h_{q^{\prime },n}^{†}. \end{array}$$

To get the scalings of the anomalous diffusion in Equations (23), (30) and (A11), we focus on the first term on the right side. In the case of the viscous media, we use $E_{1,1}\left(-t/\tau \right)=e^{-t/\tau }\Theta \left(t\right)$ to obtain Equations (23) and (30).

Next, let us see the time evolution of the paired variable $F_{q}\left(t\right)=dP_{q}\left(t\right)/dt$. Equation (A12) for $F_{q}\left(t\right)$ is transformed into the Laplace domain as

(A33) $$\begin{array}{c} m_{q}\left[\omega {\hat{F}}_{q}\left(\omega \right)-F_{q}\left(0\right)\right]=-g_{q}\hat{M}\left(\omega \right){\hat{F}}_{q}\left(\omega \right)+{\hat{V}}_{q}\left(\omega \right)+{\hat{\Xi }}_{q}^{\left(p\right)}\left(\omega \right), \end{array}$$

where $\hat{M}\left(\omega \right)≃\omega^{1-\lambda }$. The solution to Equation (A33) is obtained as

(A34) $$\begin{array}{c} {\hat{F}}_{q}\left(\omega \right)=\frac{1}{m_{q}}\frac{{\hat{V}}_{q}\left(\omega \right)+{\hat{\Xi }}_{q}^{\left(p\right)}\left(\omega \right)}{\omega +\left(g_{q}/m_{q}\right)\hat{M}\left(\omega \right)}+\frac{F_{q}\left(0\right)}{\omega +\left(g_{q}/m_{q}\right)\hat{M}\left(\omega \right)}. \end{array}$$

As performed in Equation (A31), we get

(A35) $$\begin{array}{ccc} f(t) & = & \sum_{q}\int_{0}^{t}ds\;\frac{V_{q}\left(s\right)+\Xi_{q}^{\left(p\right)}\left(s\right)}{m_{q}}E_{\lambda ,1}\left(-\frac{{\left(t-s\right)}^{\lambda }}{\left(m_{q}/g_{q}\right)\tau_{u}^{\lambda -1}}\right)\\ & & +\sum_{q}F_{q}\left(0\right)E_{\lambda ,1}\left(-\frac{t^{\lambda }}{\left(m_{q}/g_{q}\right)\tau_{u}^{\lambda -1}}\right). \end{array}$$

Using this, we caluclate the force correlations at $V_{q}\left(t\right)=0$:

(A36) $$\begin{array}{cc} & \langle \delta f\left(t\right)\delta f\left(t^{\prime }\right)\rangle \\ = & \int_{0}^{t}ds\int_{0}^{t^{\prime }}ds^{\prime }\;\sum_{q,q^{\prime }}\frac{\langle \Xi_{q}^{\left(p\right)}\left(s\right)\Xi_{q^{\prime }}^{\left(p\right)}\left(s^{\prime }\right)\rangle }{m_{q}m_{q^{\prime }}}h_{q,n}^{†}h_{q^{\prime },n}^{†}\\ & \times E_{\lambda ,1}\left(-\frac{{\left(t-s\right)}^{\lambda }}{\left(m_{q}/g_{q}\right)\tau_{u}^{\lambda -1}}\right)E_{\lambda ,1}\left(-\frac{{\left(t^{\prime }-s^{\prime }\right)}^{\lambda }}{\left(m_{q^{\prime }}/g_{q^{\prime }}\right)\tau_{u}^{\lambda -1}}\right)\\ & +\sum_{q,q^{\prime }}\langle F_{q}\left(0\right)F_{q^{\prime }}\left(0\right)\rangle h_{q,n}^{†}h_{q^{\prime },n}^{†}\\ & \times E_{\lambda ,1}\left(-\frac{t^{\lambda }}{\left(m_{q}/g_{q}\right)\tau_{u}^{\lambda -1}}\right)E_{\lambda ,1}\left(-\frac{t^{\prime \lambda }}{\left(m_{q^{\prime }}/g_{q^{\prime }}\right)\tau_{u}^{\lambda -1}}\right). \end{array}$$

The first term on the right side is a dominant factor and provides Equations (37) and (A15). To get $\langle \delta p{\left(t\right)}^{2}\rangle$, we carry out the double time integral of Equation (A36).
